# Using bamboo biochar with compost for the stabilization and phytotoxicity reduction of heavy metals in mine-contaminated soils of China

**DOI:** 10.1038/s41598-017-03045-9

**Published:** 2017-06-02

**Authors:** Amjad Ali, Di Guo, Yue Zhang, Xining Sun, Shuncheng Jiang, Zhanyu Guo, Hui Huang, Wen Liang, Ronghua Li, Zengqiang Zhang

**Affiliations:** 1 College of Natural Resources and Environment, Northwest A&F University, Yangling, 712100 China; 2 School of Resources and Environment, Anhui Agricultural University, Hefei, 230036 China

## Abstract

Anthropogenic activities have transformed the global geochemical cycling of heavy metals (HMs). Many physical, chemical and biological methods are used to reduce the toxicity of HMs to humans, plants and environment. This study aimed to investigate the immobilization and phytotoxicity reduction of HMs after application of bamboo biochar (BB) in mine-polluted soil in Feng county (FC) and Tongguan (TG). The results showed that BB application to contaminated soil immobilized HMs (Zn, Pb, Cd and Cu). The soil pH and EC increased and the bioavailability of HMs decreased in FC and TG, whereas Pb and Cu increased in TG soil. The addition of BB reduced HMs uptake in the shoot/root of *Brassica juncea*. Physiological responses showed that BB application improved the shoot/root growth, dry biomass, and enhanced the chlorophyll (*a* and *b*) and carotenoid concentrations in *Brassica*. The incorporation of BB improved the soil health and accelerated enzymatic activities (β-glucosidase, alkaline phosphatase and urease) in HMs polluted soils. Antioxidant activities (POD, PPO, CAT and SOD) were also used as biomarkers to determine the negative effects of HMs on the growth of *Brassica*. Overall, the immobilization potential and phytotoxicity reduction of HMs were confirmed by BCF, TF and MEA for both soils.

## Introduction

The rapidly growing population, industrial progress and technical innovations have increased the concentration of heavy metals (HMs) around the globe^1^. HMs pollution can be hazardous to soil, plant and human health through the soil–crop–food chain^2, 3^. The major sources of HMs are textile, energy and power, mining and smelting, coal combustion for energy purposes in large industries, agricultural practices (fertilizers and pesticides) and municipal wastes^4, 5^. The effects of HMs on plant and human health have been widely studied^6–8^. HMs enrichment affect soil enzymatic activities and the biosynthesis of chlorophyll, decrease respiration and limit antioxidant enzymatic activities, and even lipid peroxidation in plant cells^9^. The enzymatic activities of plant i.e. guaiacol peroxidase (POD), polyphenol oxidase (PPO), catalase (CAT) and superoxide dismutase (SOD) are widely used as sensitive biomarkers in HMs contaminated soil^10, 11^.

Numerous amendments are used to immobilize HMs and reduce their bioavailability to facilitate the establishment of plants in HMs-polluted soil. They include, CaO, phosphate fertilizers, fly ash, medical stone, organic waste (compost, crop residues and bio-solid compost), zeolite and biochar^12–14^. Among these practices, biochar is widely used for HMs immobilization in polluted soil^15^. Biochar application is an old practice originating from slash-and-burn agriculture, which may be a method to limit HMs (Zn, Cd, and Pb) mobility from the soil to living matrices^16, 17^. Medical stone is widely used in the medical care and purification of polluted water due to a high cation exchange capacity (CEC), porous structure and large surface areas. However, there is limited research on the potential effects of medical stone added pig manure compost (MSC) and biochar in reducing the bioavailability of HMs in mine soil^14, 18^.

Biochar is a carbon-rich biological charcoal, produced under slow pyrolysis (limited oxygen) of organic residues (crop straw, animal litter, wood chips) at a high temperature^10, 19^. Biochar amendment can improve soil fertility and plant productivity by improving the soil physico-chemical and biological properties^11, 18^. Biochar has a high surface area, nutrient and water holding capacity, resists decomposition in soil and retains specific effects for a longer time. Biochar has alkaline pH, high CEC and enhanced carbon sequestration, making it an efficient adsorbent for soil HMs, mitigating climate changes by reducing CH_4_ emission and reducing environmental pollution^20, 21^. Biochar has been reported to enhance the defense mechanism in plants by improving the plant’s antioxidant enzymatic activities^10, 11^. The stabilization of HMs in polluted soil is an appealing research area and promising technology for restoring degraded soils and reducing phytotoxicity. However, the remediation process of mining sites is influenced by the presence of a variety of HMs and soil conditions^22^.

*Brassica juncea* is a member of the *Brassicaceae* family. Brassica is a promising hyperaccumulator and fast-growing plant, used for the phytoremediation of contaminated soil. *Brassica* is commonly cultivated in the study areas for oil production and forage purpose. It has the capacity to accumulate huge amount of HMs in its biomass^5^. Biochar and brassica are widely studied for the translocation assay of HMs in shoots and roots^23^. This study aimed to immobilize HMs and reduce their phytotoxicity in smelter/mine polluted soil of Feng county (FC) and Tongguan (TG) through the addition of bamboo biochar (BB) and MSC. The effect of BB was compared in terms of phytoextraction indices.

## Results and Discussion

The basic physico-chemical characteristics of the FC, TG soil, MSC and BB are shown in Table 1.

**Table 1:** Main characteristics of mine contaminated soils, compost and bamboo biochar.

Soil	FC soil	TG Soil	Compost	Bamboo Biochar
pH	7.72	8.06	6.57	8.86
EC_e_ (µS cm^−1^)	422	201.3	732.3	537.36
Clay %	1.56	0.50	—	—
Silt %	48.43	22.10	—	—
Sand %	50.01	77.33	—	—
Soil texture	Sandy loam	Loamy sand	—	—
CEC (cmol^+^ kg^−1^)	23.5	19.5	210.5	14.25
Total Nitrogen (g kg^−1^)	1.23	0.689	15.64	7.13
Total Phosphorus (g kg^−1^)	0.848	0.861	22.50	2.07
Total Potassium (g kg^−1^)	19.50	10.30	13.25	6.56
Total organic carbon (g kg^−1^)	8.64	16.47	385.73	730
BET (m^2^ g^−1^)	—	—	—	235.3
Total HMs in soil (Feng county and Tongguan), compost and bamboo biochar (mg kg^−1^)				
Zn	6625	230.4	384.54	18.63
Pb	204.4	393.2	8.09	0.06
Cd	117.7	1.58	0.557	BDL
Cu	51.1	141.3	77.42	12.17
Al	31629	40529	—	—
As	11.1	14.5	—	14.76
Ca	15538	24938	—	—
Co	16.20	11.95	—	—
Cr	61.75	52.54	—	—
Fe	25694	19215	—	—
Hg	0.30	0.783	0.55	—
Mg	8205	8592	—	—
Mn	729.7	545.5	—	—
Mo	0.85	0.908	—	—
Na (mg kg^−1^)	9200	10574	—	—
Extractable HMs in soil (Feng county and Tongguan) and compost (mg kg^−1^)				
Zn	584	15.59	171.11	—
Pb	40.2	200.0	5.50	—
Cd	36.4	0.549	0.30	—
Cu	1.77	11.65	20.56	—

*Values indicate mean of one sample with three replications.

BDL indicates values below detection limit.

### Effect of biochar amendments on soil pH and EC

**Figure 1:**
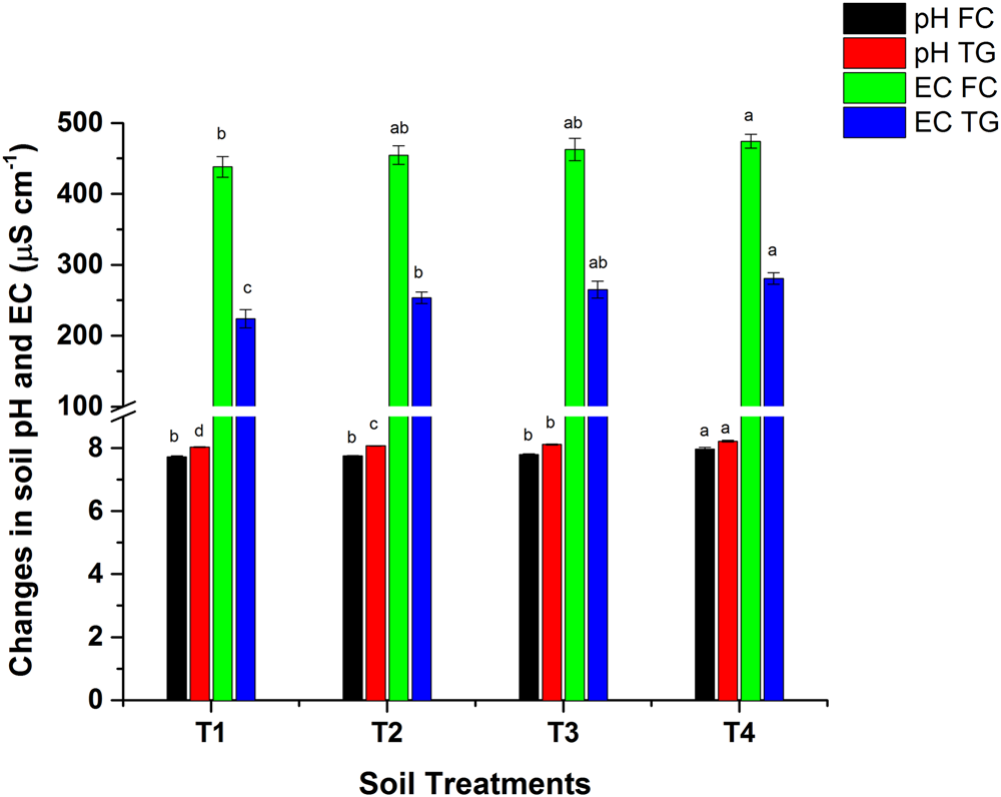
Effect of bamboo biochar amendments on soil pH and EC in Feng county and Tongguan soil. The data represent the mean of three replicates, and the error bars are standard deviations. Means with different letters are significantly different (*p* < 0.05). (T1 = Control, T2 = 1.0% BB, T3 = 2.5% BB, T4 = 5.0% BB).

Biochar releases cations into the soil after addition, which can slightly raise the pH and EC. This can facilitate HMs bioavailability to the plants and can be translocated into the shoot, especially in acidic soil^20, 24^. The impact of biochar application on soil pH and EC values is shown in Fig. 1. A significant (*p* < 0.05) increase in soil pH and EC was recorded after the addition of BB. The release of alkali salts from the feedstock (bamboo) during pyrolysis increase soil pH^25, 26^. The highest mean values of soil pH and EC were reported in T5 (5% BB) pots, compared to their respective controls in both the FC and TG soils. The soil pH increased from 7.72–7.96 and 8.03–8.21, whereas EC increased from 438.25–474.33 and 224–280.67 µS cm^−1^ in the FC and TG soil, respectively. The soil pH increased by an average of 0.24 and 0.18 units, whereas EC increased by 36 and 56.6 units at a 5% BB application to smelter (FC) and mines (TG) polluted soils, respectively. These results were in accordance with the previous research findings^24, 27^.

### Effect of biochar amendments on HMs bioavailability

**Figure 2:**
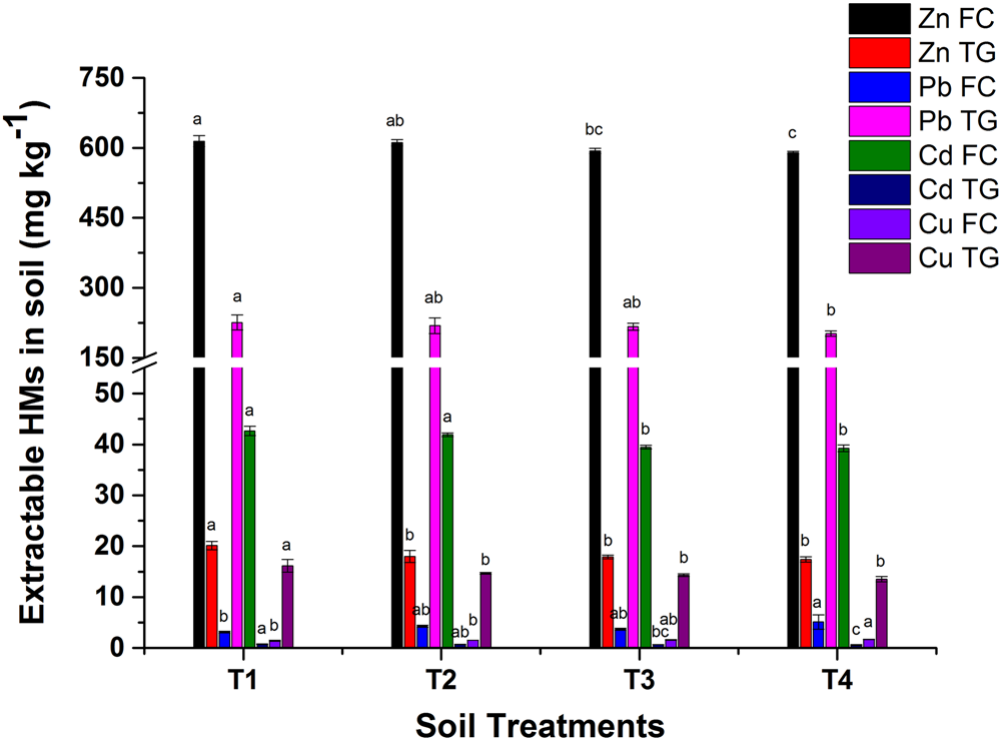
Effect of bamboo biochar amendments on extractable HMs in Feng county and Tongguan. The data represent the mean of three replicates, and the error bars are standard deviations. Means with different letters are significantly different (*p* < 0.05). (T1 = Control, T2 = 1.0% BB, T3 = 2.5% BB, T4 = 5.0% BB).

DTPA extractable heavy metals are considered as available to plants in the soil system. Biochar can immobilize HMs by precipitates formation (phosphates), the adsorption of HMs, electro-static interaction, ionic exchange between metal cations and biochar derived protons as well as the formation of stable chelates and complexes with organic matter^25, 28^. The availability of Zn, Pb, Cu and Cd decreased significantly (*p* < 0.05) with BB amendments and was more distinct at 2.5 and 5% application rates. The effect of BB on the bioavailable fraction of HMs in FC and TG soil is shown in the Fig. 2. The results showed that the bioavailability of Zn and Cd decreased by 4 and 8%, whereas the concentration of Pb and Cu increased by 65 and 17%, respectively in T4 (5% BB) in the case of FC soil. For the organic source of nutrition, 2.5% MSC was added to each pot, which contained traces of Pb and Cu^14^. Inactivated BB can promote dissolved organic matter (DOM), which may form soluble Pb and Cu complexes^15, 16^. The HMs concentration is far higher in FC than in TG soil. The higher concentration of HMs in FC soil limited the stabilization efficiency of BB. DTPA-extractable HMs reduced in the following order: Cd (8%) >Zn (4%), whereas Pb (65%) and Cu (17%) increased in FC soil.

The bioavailable concentration of Zn, Pb, Cd and Cu decreased by 14, 11, 23 and 16%, respectively in TG soil. Similarly, DTPA extractable Cd, Pb, and Zn were reduced by 90, 38, and 24%, respectively, after 10% orchard prunes biochar was incorporated with mine tailings^29^. DTPA-extractable Pb by decreased 17.5% and 16.6% in the tillering stage and maturity stage in rice, respectively^10^. Our findings were consistent with another study, 5% rice straw biochar reduced extractable Cu and Zn by 97.3 and 62.2%, respectively^28^. The higher adsorptive capacity of biochar helps in the stabilization and immobilization of HMs. Biochar addition modifies CEC and soil pH, providing favorable conditions for HMs immobilization and lower phytoavailability to reduce phytotoxicity^11, 24^. The presence of phenolic, -OH, -COOH and C=N groups on BB showed stronger adsorption of HMs^10, 25^. Similarly, DTPA-extractable HMs decreased in the following order in TG soil: Cd (23%) > Cu (16%) > Zn (14%) > Pb (11%).

### Effect of biochar on HMs translocation in *Brassica juncea* shoot

**Figure 3:**
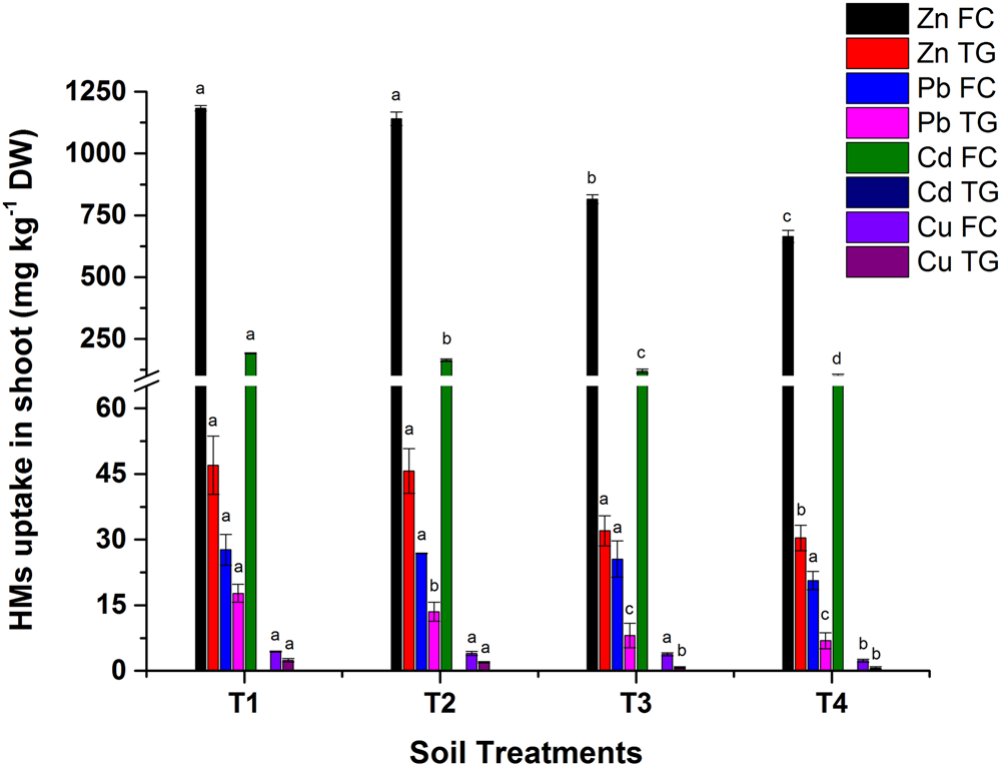
Effect of bamboo biochar on shoot uptake (mg kg^−1^ DW) of HMs in *Brassica juncea*. The data represent the mean of three replicates, and the error bars are standard deviation. Means with different letters are significantly different (*p* < 0.05). (T1 = Control, T2 = 1.0% BB, T3 = 2.5% BB, T4 = 5.0% BB).

Biochar improved growth and reduced the availability and mobility of HMs in plant tissues. Soil pH is of vital importance to decreasing bioavailable HMs in soil. BB is alkaline in nature (Table 1), having a liming effect on FC and TG soil^27, 28^. The content of HMs (Zn, Pb, Cd and Cu) translocated in the *Brassica juncea* shoot is shown in Fig. 3. The significant (*p < *0.05) reduction in the bioavailable concentration of HMs in soil resulted in low translocation in the shoot of *Brassica* grown in FC and TG soil. Maximum of 44, 25, 48 and 47% decreases in Zn, Pb, Cd and Cu were reported for a 5% BB application rate (T4) in FC soil. Similarly, another study reported 33 and 75% decreases in Cd and Pb uptake in shoots after 5 and 10% biochar incorporation in soil, respectively^26^. Likewise, Cd and Pb uptake in *Nicotiana tabacum* significantly decreased by 5.4–52.3 and 7.2–58.5%, respectively^27^.

The uptake diminution was 35, 61 and 72% for Zn, Pb and Cu in TG soil, respectively. Meanwhile, Cd content was below detection limit in *Brassica* grown in TG soil due to very low bioavailable fraction. Our results implicated that the HMs uptake in mines polluted soil was reduced after the BB application at different rates. Biochar transform the readily available fraction of HMs into geochemically stable fraction, resulting in reduced mobility and bioavailability of HMs^20, 24, 29^. Similarly, bamboo biochar reduced the extractable Cu by 31.9 and 66% at 1 and 5% rate, respectively^28^. Biochar is considered more appropriate for the remediation of organic and inorganic pollutants (HMs) due to its ability to limit their translocation into plant shoots. The HMs stabilization and sorption onto bamboo biochar might be attributed to ion exchange, precipitate formation of HMs carbonates and phosphatase, chemisorption, complexation as well as surface interaction^30, 31^.

### Effect of biochar on HMs accumulation in *Brassica juncea* root

**Figure 4:**
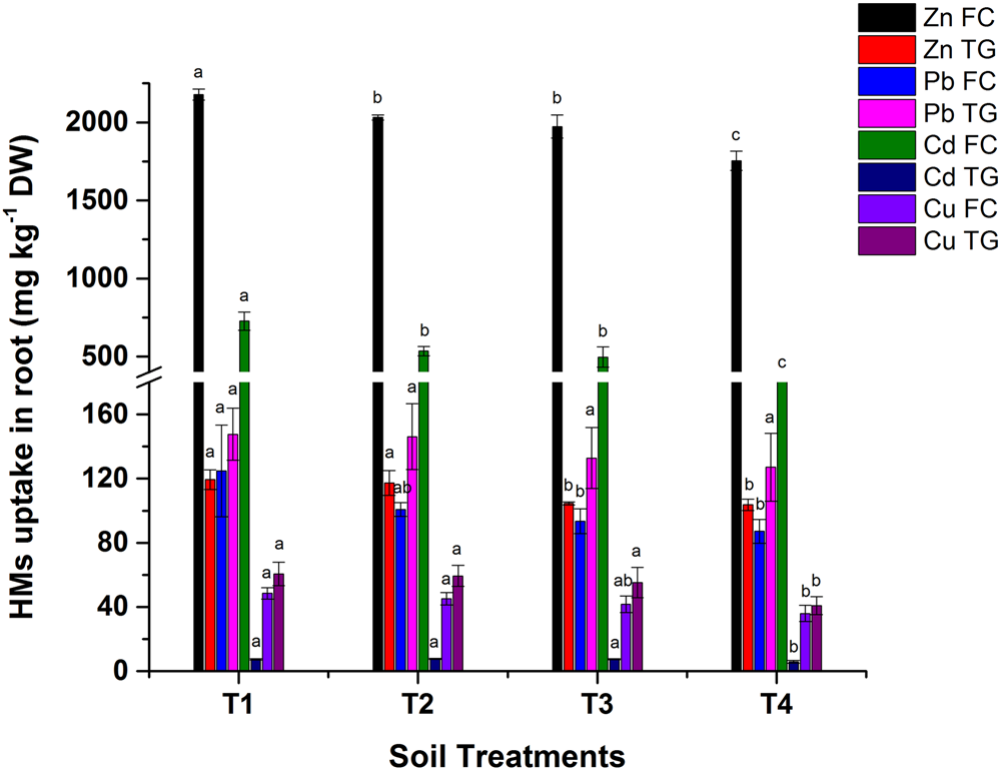
Effect of bamboo biochar on root uptake (mg kg^−1^ DW) of TEs in *Brassica juncea*. The data represent the mean of three replicates, and the error bars are standard deviations. Means with different letters are significantly different (*p* < 0.05). (T1 = Control, T2 = 1.0% BB, T3 = 2.5% BB, T4 = 5.0% BB).

The HMs translocation in *Brassica* root grown in FC and TG soil is shown in Fig. 4. Data revealed that HMs uptake in roots were significantly (*p* < 0.05) influenced by biochar application and followed the same diminution trend as in shoot HMs. Biochar has a high adsorption capacity and facilitates the fixation of HMs in polluted soil. The root uptake of Zn, Pb, Cd and Cu was reduced by as much as 19%, 30%, 50 and 28%, respectively, in T4 in the case of FC soil and 13%, 14%, 19 and 32%, respectively, in TG soil compared to their respective controls. Similarly, 28, 60 and 53.2% reductions in Zn, Cu and Cd, respectively were reported after 10% biochar application to soil^24^. These results further illustrated that the reduction in the bioavailable fraction in FC and TG soil decreased the uptake of HMs in *Brassica* roots.

Another study reported as high as 55–78, 29–50 and 11–46% decreases in Zn, Pb and Cd, respectively after 90 days of incubation^13^. The soil pH increased after the application of BB in FC and TG soil. The increase in pH provided more negatively charged surfaces (-OH, and -COOH) in soil^25^. This might have increased the sorption capacity of FC and TG soil for cationic metals such as Zn, Pb, Cd and Cu^32^. HMs adsorb on the biochar surface and can make complex with the dissolved organic C and HCO_3_ in biochar. This will lead to immobilization of HMs, reduce their concentration in soil and lower translocation in plants^27, 33^.

### Shoot and root dry biomass

**Figure 5:**
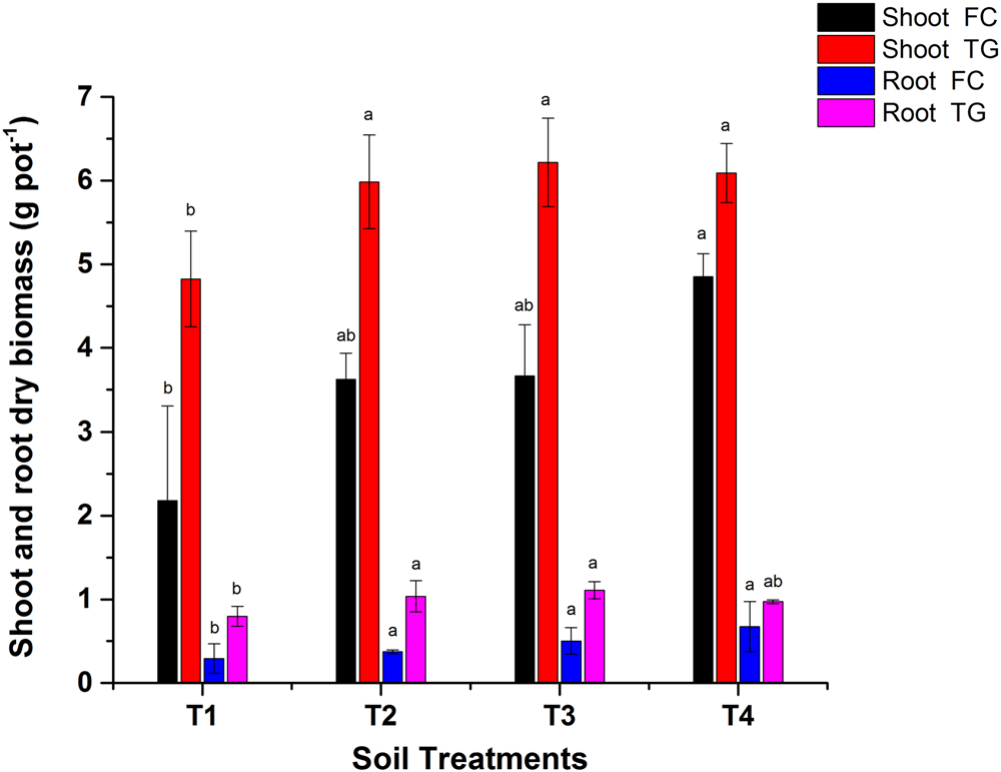
Effect of bamboo biochar on shoot and root dry biomass (g pot^−1^) of *Brassica juncea*. The data represent the mean of three replicates, and the error bars are standard deviations. Means with different letters are significantly different (*p* < 0.05). (T1 = Control, T2 = 1.0% BB, T3 = 2.5% BB, T4 = 5.0% BB).

The incorporation of biochar in soil improved the growth and yield as well as increased the photosynthetic rate in plant^11^. The application of BB to FC and TG contaminated soil significantly (*p* < 0.05) increased the shoot and root dry biomass of *Brassica* compared to their respective controls (Fig. 5). Maximum of 123 and 131% increases in the shoot and root dry biomass of FC were reported in T4 (5% BB). Other researchers also reported the role of biochar in enhancing the dry weight of roots, stems and leaves of plants^10, 26^. The root dry biomass of *Nicotiana tabacum* increased by 63.9–128.2% compared to the control^27^.

The shoot and root biomass were increased by 29 and 39% in T3 (2.5% BB) in *Brassica* grown in TG soil. The results of the TG soil showed that the application of a higher BB dose (5%) limited the shoot and root biomass. In contrast to the results obtained in TG soil, rice straw and conocarpus biochar have been reported to increase the shoot dry biomass of *Nicotiana tabacum* and *Zea mays* by 56.1–90.7% and 54.5–102%, respectively^24, 27^. In addition, dry biomass of *Zea mays* and fresh weight of *Malus hupehensis* were improved by 101 and 100% after 5 and 8% biochar amendment^8, 11^. Our findings are also supported by Lu, *et al*.^17^, who reported 3.44 and 2.24 times increases in the total plant biomass after poultry and eucalyptus biochar addition to Cd polluted soil.

### Chlorophyll and carotenoid content in *Brassica juncea* leaves

**Figure 6:**
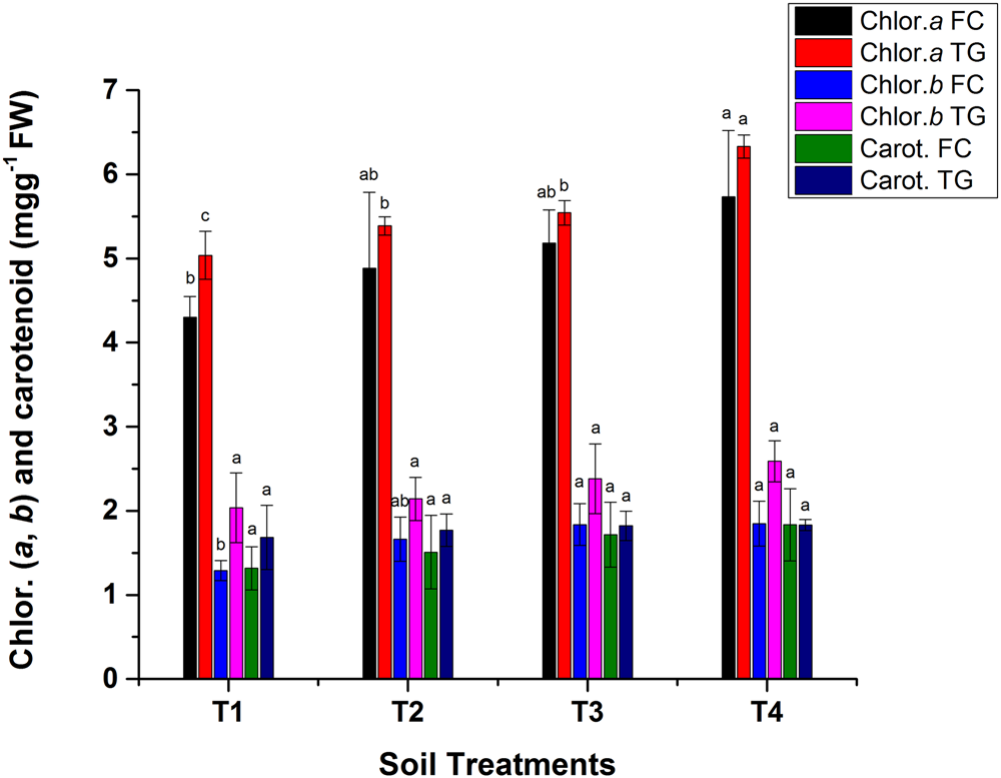
Effect of bamboo biochar on chlorophyll (*a* and *b*) and carotenoid (mg g^−1^ FW) in *Brassica juncea*. The data represent the mean of three replicates, and error bars are standard deviations. Means with different letters are significantly different (*p* < 0.05). (T1 = Control, T2 = 1.0% BB, T3 = 2.5% BB, T4 = 5.0% BB).

HMs induced ROS (reactive oxygen species) impair the biosynthesis of chlorophyll and carotene in plant cells, which can lead to leaf chlorosis^9, 13^. The application of bamboo biochar significantly increased chlorophyll (*a* and *b*) and the effect on the carotenoid content in *Brassica* leaves was not significant (*p < *0.05). HMs adversely affect the chlorophyll pigments in plants. However, these pigments are improved by the release of N and P from biochar^5^. Chlorophyll pigment data for *Brassica* are shown in Fig. 6. Chlorophyll (*a*, *b*) and the carotenoid content in *Brassica* grown in FC soil improved by 33.4, 43.2 and 39.2% in T4, as compared to untreated pots. The leaf enzymatic activities were enhanced after BB application, which can alleviate the ROS stress posed by HMs. Hence, the chlorophyll pigments were enhanced after the addition of biochar^11, 34^.

Meanwhile, 5% BB application enhanced the chlorophyll (*a*, *b*) and carotenoid in *Brassica* by 26, 27 and 9%, respectively in TG soil. The increase in chlorophyll pigments in the TG samples was comparatively lower than in the FC samples. The bioavailable HMs in FC soil were high, which might have damaged the biosynthesis of chlorophyll pigments in plant cells. This could have impaired the uptake of Mg and Fe in *Brassica* leaf^35^. The application of BB reduced the HMs stress in polluted soils by stabilization and the chlorophyll contents (a and b) in *Brassica* leaves were higher in the pots receiving biochar compared to control. Similarly, another study reported 16, 25.4 and 31.5% increases in the chlorophyll content in *Malus hupehensis* after 5, 20, and 80 g kg^−1^ biochar, respectively^11^.

### Effect of biochar on the soil enzymatic activities

**Figure 7:**
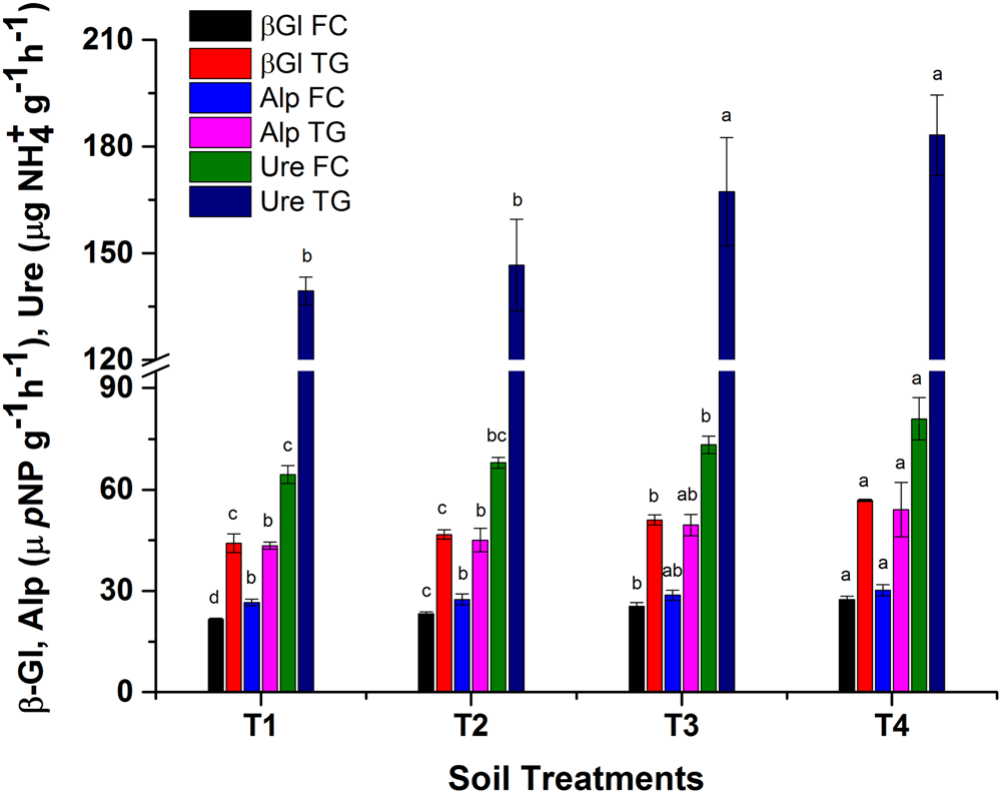
Effect of bamboo biochar on the soil enzymatic activities of Feng county and Tongguan. The data represent the mean of three replicates, and error bars are standard deviations. Means with different letters are significantly different (*p* < 0.05). (T1 = Control, T2 = 1.0% BB, T3 = 2.5% BB, T4 = 5.0% BB).

Soil enzymatic activity is an important indicator of soil health in monitoring the effect of HMs contamination, soil management and agriculture practices^15, 28^. The effect of BB on β-glucosidase, alkaline phosphatase and urease activities were assayed and presented in Fig. 7. Our results showed that the enzymatic activities in mines polluted FC and TG soil were significantly (*p* < 0.05) enhanced. This might be due to the supply of carbon and essential nutrients by BB^36^. Biochar application increased β-glucosidase, phosphatase and urease activities by 27.31 and 29%, 14 and 25% as well as 26 and 31.44% in FC and TG soil, respectively. The effects of lower doses of biochar were also significant compared to their respective control pots.

β-glucosidase releases low molecular weight sugars, which is an energy source for soil microbes and plays a vital role in the global C cycle^37^. Biochar is a carbon rich source that effectively improves the β-glucosidase activities in both soils. The soil (FC and TG) and biochar are alkaline, which favored alkaline phosphatase activity. Alkaline phosphatase helps in P mineralization in soil and root development. The presence of phosphorus in the MSC and BB can explain the mechanism of alkaline phosphatase activities. Biochar can affect soil microorganisms involved in the nutrient transformations and cycling^38^. Urease assists the nutrients and organic matter transformation in soil. Urease activity was enhanced by BB. Urease activity was amplified by 143% with 5% rice straw biochar incorporation^28^. The higher urease and alkaline phosphatase activities are ascribed to the higher nitrogen and phosphorus content in MSC and BB^39^. Previous scientific reports have also revealed the role of biochar in enhancing β-glucosidase, alkaline phosphatase and urease activities in polluted soil^7, 18^. Our findings showed a positive effect of BB on soil enzymatic activities in mine-contaminated soil.

### Effect of biochar on the plant antioxidant enzymes

**Figure 8:**
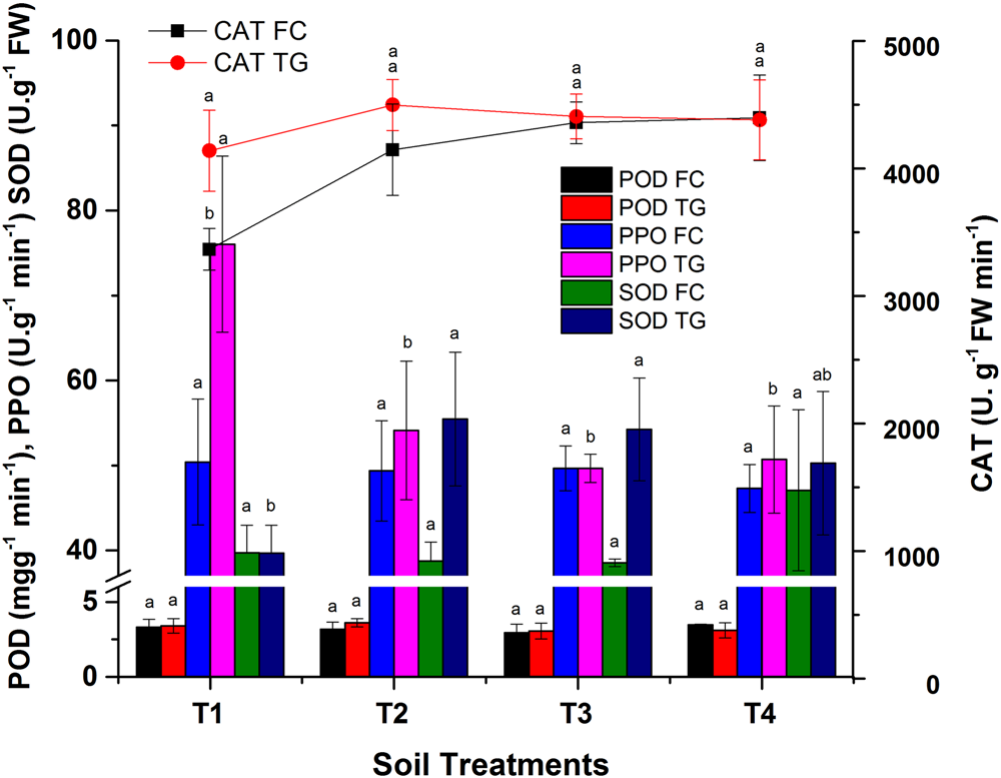
Effect of bamboo biochar on leaf antioxidant enzymatic activities in *B. juncea*. POD was expressed in mg g^−1^ min^−1^, PPO in U. g^−1^ min^−1^, CAT in U. g^−1^ FW min^−1^ and SOD U g^−1^ FW. The data represent the mean of three replicates, and error bars are standard deviations. Means with different letters are significantly different (*p* < 0.05). (T1 = Control, T2 = 1.0% BB, T3 = 2.5% BB, T4 = 5.0% BB).

Abiotic and biotic plant stress can lead to ROS (O2^•−^, H_2_O_2_ and OH^·^) formation through different pathways. Excessive formation of ROS leads to oxidative stress, DNA damage, membrane permeability, loss of cell function and even cell death^40^. Plants develop antioxidant enzymatic activities (POD, PPO, CAT and SOD) to scavenge ROS and avoid or increase resistance against stress^11^. The application of BB affects antioxidant enzymatic activities (Fig. 8), which can improve the detoxification of HMs by plants.

In stress conditions, POD converts H_2_O_2_ into H_2_O and O_2_ by catalysis^10^. POD activity can be used as a biomarker for HMs toxicity in *Brassica*. POD activities decreased in *Brassica* grown in FC and TG soil. A maximum of 12 and 10% decline was observed in T3 (2.5% BB) in FC and TG soil, respectively. Meanwhile 5 and 6% increases in POD activity were reported in T4 (TG) and T2 (FC), compared to their respective controls. Similarly, another study reported significant (*p* < 0.05) increases of 18 and 77% at 0.5 and 8% biochar amendments, respectively^11^. It is observed that higher doses of BB reduced the bioavailability of HMs, which minimized plant stress^13^. Biochar proved to be an effective amendment to reduce plant stress and facilitate the establishment of *Brassica* in the smelter/mine-polluted soil of FC and TG. Induced POD might be due to the protective measurements adopted by *Brassica* against oxidative damage pronounced by ROS. The level of POD was also reported to decline in tomato leaves with increasing biochar rates^10^.

PPO is involved in respiratory metabolism, converts phenols to chinone in plants and is known as a biomarker for HMs-induced oxidative stress^2^. The application of bamboo biochar to FC and TG soil resulted a diminution of PPO activities in *Brassica* leaves compared to their respective controls. The stress reduction might be lower in FC soil than TG soil after BB addition due to the higher degree of metal pollution in FC. Maximum of 6 and 35% diminution were observed in T4 and T3 of FC and TG soil, respectively. Biochar not only reduces the bioavailability of the HMs but also improves the plant growth/development and induces systematic resistance to metal stress^10, 33^.

CAT removes toxic peroxides in plant cells^2^. CAT activities in FC and TG soil were also improved by BB application. CAT activity in *Brassica* was augmented by 31 and 9% in FC and TG soil after the application of 5 and 1% BB (T4 and T2), respectively. The increasing CAT levels in T2-T4 can be assumed to be an adaptive mechanism developed by *Brassica* in the smelter polluted FC soil^41^. Reduction of CAT activity (T5) at higher content of HMs might be attributed to the inactivation of an enzyme by ROS, decrease in enzyme synthesis, or change in the assembly of its subunits. Biochar improved CAT activity by 38.79% in *Chlorella vulgaris* ^2^.

SOD activities have been correlated with increased tolerance to chemical and physical stress^2^. SOD activities increased in both soils with some exceptions in T2 and T3 in FC soil. SOD activity decreased in T2 and T3 (1 and 2.5% BB) and improved by 18.5% in T4 (5% BB) in FC soil. Similarly, SOD levels declined in tomato leaves after increasing the biochar dose^10^. This showed the positive effect of higher dose of BB in reducing the HMs stress in smelter/mines polluted soil. Likewise, other researchers also reported 34.18 and 12% increase in SOD activity in *Chlorella vulgaris* and *Malus hupehensis*, respectively^2, 11^. The SOD activities in *Brassica* grown in TG soil increased by 40% in T2. Induced SOD activity is ascribed to the increased production of ROS or the protective measures adopted by *Brassica* against oxidative damage. Our findings indicated that BB enhanced the antioxidant (POD, PPO, CAT and SOD) capability and alleviated HMs stress in *Brassica juncea*. The reduced antioxidant enzyme activities can be associated to the HMs immobilization effect of BB followed by decreased metal translocation into plant tissues. This led to stress reduction at a cellular level in *Brassica* ^13^.

### Phytoextraction indices of HMs

BCF, TF and MEA were used to measure the phytotoxicity reduction of bamboo biochar in smelter and mine-contaminated soil of FC and TG. The phytoextraction indices of *Brassica* are shown in Table 2. The data revealed that the BCF of HMs (Zn, Pb, Cd and Cu) decreased in biochar-treated pots of FC and TG soil (except Cd) compared to untreated control pots. This can better explain that HMs translocation in the shoot were reduced, ultimately lowering the phytotoxic effects on *Brassica*. In FC soil, the BCF value for Cd was higher than the critical value (1.0) for a hyperaccumulator plant. After biochar incorporation, it dropped from 1.63 to 0.85. HMs present in the soil were mostly adsorbed on the surface of biochar^24, 30^.

**Table 2:** Effect of bamboo biochar on the Bioconcentration Factor (BCF), Translocation Factor (TF) and Metal Extraction Amount (MEA) of *Brassica juncea* grown in FC and TG soil.

Trt	Zn	Pb	Cd	Cu	Zn	Pb	Cd	Cu	Zn	Pb	Cd	Cu
	**BCF (FC Shoot)**				**TF (FC)**				**MEA FC (µg plant** ^−**1**^ **)**			
T1	0.18	0.14	1.63	0.09	0.54	0.22	0.26	0.09	257.63	6.03	41.83	0.96
T2	0.17	0.13	1.39	0.08	0.56	0.27	0.31	0.09	413.44	9.74	59.24	1.44
T3	0.12	0.13	1.02	0.07	0.41	0.27	0.24	0.09	298.77	9.37	44.01	1.39
T4	0.10	0.10	0.85	0.05	0.38	0.24	0.28	0.06	322.49	10.03	48.68	1.13
	**BCF (TG Shoot)**				**TF (TG)**				**MEA TG (µg plant** ^−**1**^ **)**			
T1	0.20	0.05	0	0.02	0.39	0.12	0	0.04	22.68	8.54	0	1.17
T2	0.20	0.03	0	0.01	0.39	0.09	0	0.03	27.34	8.10	0	1.16
T3	0.14	0.02	0	0.01	0.31	0.06	0	0.01	19.91	5.03	0	0.48
T4	0.13	0.02	0	0.00	0.29	0.05	0	0.02	18.51	4.19	0	0.41

*Values indicate mean of one sample with three replications.

(T1 = Control, T2 = 1.0% BB, T3 = 2.5% BB, T4 = 5.0% BB).

Similarly, the TF of HMs (Zn, Pb and Cu) also reduced in a linear mode as per increasing levels of biochar incorporation in FC and TG soil. These findings are in accordance with the fact that biochar reduces the bioavailability of HMs in soil and lowers their uptake in the shoot^10, 20^. TF for Zn was higher than other HMs due to its high bioavailable concentration in both soils. The MEA of Zn, Pb, Cd and Cu showed an increase in FC soil and decrease in TG soil, except Cd. The comparatively higher MEA values were reported in FC soil due to greater increase in the shoot dry biomass of *Brassica* (Fig. 5) and higher concentration of bioavailable HMs (Table 1). Similarly, MEA in the root and shoot of *Amaranth tricolor* also decreased after the addition of poultry and eucalyptus biochar^17^. The Cd uptake in shoot was negligible in TG soil (Fig. 3). The BCF, TF and MEA values for TG soil were also negligible (zero) due to the presence of low available Cd (0.549 mg g^−1^). Overall, BCF, TF and MEA also confirmed the adsorption potential of bamboo biochar to lower HMs translocation and reduce its phytotoxicity in mine-polluted soil of FC and TG.

## Conclusions

The results show that BB application to smelter and mine-contaminated soil immobilized HMs (Zn, Pb, Cd and Cu). The application of BB increased the soil pH and EC to form insoluble metal complexes/precipitates (phosphates) in soil and reduced the bioavailability of HMs in FC and TG soil, except Pb and Cu in TG soil. BB reduced the HMs uptake in the *Brassica* shoot/root and lowered the phytotoxicity. The physiological responses of *Brassica* showed that BB application improved plant growth, increased the shoot/root dry biomass, and augmented the chlorophyll (*a* and *b*) and carotenoid contents. The incorporation of BB improved the soil health and enhanced the enzymatic activities (β-glucosidase, alkaline phosphatase and urease) in HMs-polluted soils. Furthermore, the plant antioxidant activities (POD, PPO, CAT and SOD) were also used as natural biomarkers to measure the HMs stress in *Brassica*. BB application in mine-polluted soil resisted the oxidative stress in HMs-polluted soils. BB application in FC and TG soil improved the immobilization potential and reduced the phytotoxicity of HMs, which was also confirmed by the BCF, TF and MEA results.

## Materials and Methods

### Materials collection

**Figure 9:**
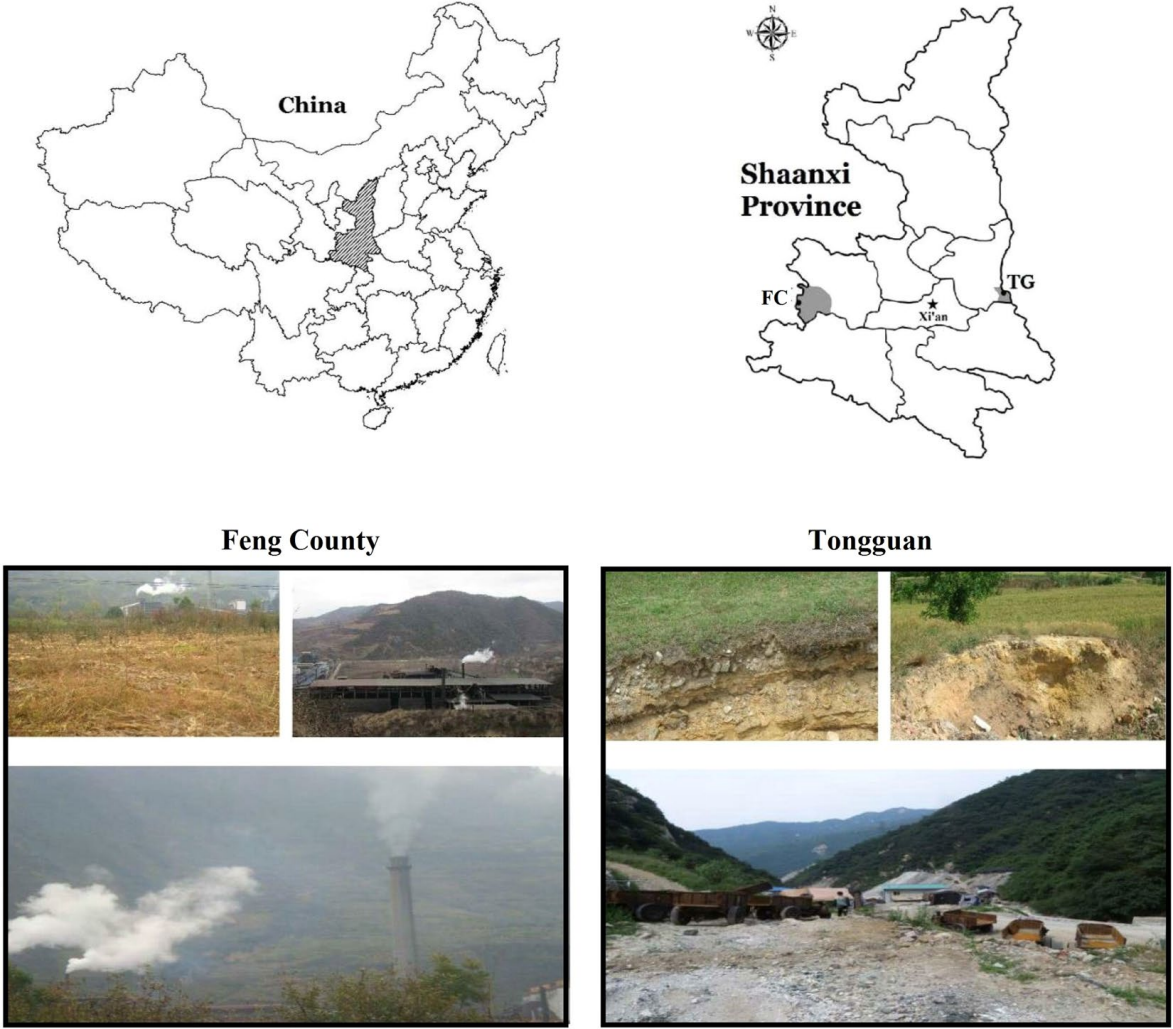
Sampling sites in Feng County and Tongguan, Shaanxi province, China. The map was adopted from Shen, *et al*.^42^ with permission. The maps were created using ESRI’s ArcGIS 9.0 software (http://www.esri.com/software/arcgis).

Smelter and mine contaminated soils were collected from Feng County (106°24′~106°35′N, 33°34′~34°56′E) and Tongguan (34°27′~34°37′N, 110°10′~110°23′E), respectively, in Shaanxi province, China (Fig. 9). Both locations have a long history of smelting and mining activities, which can limit the agricultural productivity. Remediation of such sites is necessary for environmental cleanup and increasing crop yield. The Feng county, located in the southwest of Shaanxi province, is one of the largest mining sites, with 4.5 million tons of Zn/Pb reserves. The prime pollution sources are mine wastewater, atmospheric deposition and mine tailings^21^. Tongguan is a famous gold mining site in Shaanxi province. The area is mainly polluted with mining, mineral processing and atmospheric deposition of HMs^12^. Large area (50 × 50 m^2^) in FC and TG site was selected for soil sampling near the source of pollution. Soil samples were collected from small segments (25 m^2^) and mixed to form a composite sample. The contaminated soil samples were collected from 0–20 cm soil depths. The composite contaminated soil samples were stored in polyethylene bags and immediately transferred to the laboratory. The soils samples collected from both locations were air-dried in shade at room temperature, crushed manually and then passed through 2 mm sieve. The samples were stored and used in the experiments, applied with different levels bamboo biochar and MSC. Bamboo biochar was purchased from Shaanxi Yixin Energy Company, Yangling, China. The medical stone was purchased from Shijiazhuang Building Materials Co. Ltd., China. Pig manure and sawdust were collected from a local pig farm and wood-processing factory in Yangling, China.

## Experimental Methods

### Composting and pot trial for *Brassica juncea*

To prepare the MSC, medical stone (2.5%) was added to pig manure and mixed with sawdust (2:1 dry weight bases) in a 130 L PVC composter for 45 days^10, 14^. The mature MSC was used in the experiment. A pot experiment for the two soils (FC and TG) was laid out in a complete randomized design under a mobile shelter house in an open environment. Four treatments for each soil were performed i.e. T1 (Control), T2 (1.0% BB), T3 (2.5% BB), and T4 (5.0% BB). The pots (15 × 12 × 9 cm^3^ dimension) were filled with one kg (1000 g) homogenized soil as growing media. MSC (2.5%) was added as a soil conditioner and nutritional supplement to each pot. The seeds of *Brassica juncea* (ShaanYou 16) were sterilized in 3% H_2_O_2_ and ten seeds per treatment were grown in triplicates. The dry biomass of the *Brassica* shoot and root were recorded after 7 weeks.

### Analytical methods for soil, compost and bamboo biochar

Basic characteristics, such as the like soil pH (1:2), electrical conductivity (EC) and organic matter were measured by standard methods^10^. The soil particle size distribution and cation exchange capacity were estimated as described by Mahar *et al*.^12^. Total N and P were determined according to the Kjeldahl and molybdenum antimony blue colorimetry methods, respectively^6^. The total HMs in FC and TG soil, bamboo biochar and MSC were measured by ICP-AES^43^. Soil DTPA/TEA extractable HMs (Zn, Pb, Cd and Cu) were tested according to Lu *et al*.^17^. The Brunauer–Emmett–Teller (BET) surface area of bamboo biochar was determined by N_2_ sorption analysis at 77 K in a surface analyzer (TristarII 3020, Micromeritica Instrument Corporation, USA). All chemicals of analytical grade were prepared in double deionized water for analytical purposes^12^.

### Phytotoxicity assay of *Brassica juncea* in polluted soils

For the phytotoxicity assay, the effect of BB on shoot/root dry biomass, chlorophyll (*a* and *b*) and carotenoid content in *Brassica*, grown in FC and TG soil were tested.

### Determination of photosynthetic pigments content

The chlorophyll (*a* and *b*) and carotenoid contents in *Brassica* leaves were extracted and assayed. Simply, we dissolved 0.2 g grinded *Brassica* leaf in 80% acetone for 24 hours in dark and measured the absorbance at 663, 645 and 470 nm for the Chlor *a*, Chlor *b* and carotenoid contents, respectively^11^. The pigment concentration was expressed in mg g^−1^ FW.

### Determination of total heavy metals in plant samples

After 7 weeks, *Brassica juncea* was harvested and separated into the shoot and root. The shoot and root samples were washed with tap water followed by rinsing with deionized water. The samples were dried to a constant weight at 60–70 °C. The dried samples were crushed into fine powder prior to the analysis. The *Brassica* shoot (0.50 g) and root (0.25 g) samples were digested with HNO_3_–HClO_4_ (3:1) to determine the total concentrations of HMs^44^.

### Determination of soil and plant enzymatic activities

To detect the effect of BB on the enzymatic activities of soil; β-glucosidase^7^, alkaline phosphatase^9^ and urease^28^ activities were determined in the soil after harvesting *Brassica juncea*. Plant antioxidant activities i.e. Guaiacol peroxidase (POD), Polyphenol oxidase (PPO), catalase (CAT) and superoxide dismutase (SOD) activities were also assayed according to standard procedures^10, 11, 45^. Briefly, POD activity was assayed by changes in absorbance at 470 nm. PPO activity was assayed by measuring the increase in absorbance at 370 nm. CAT activity was measured by changes in absorbance at 240 nm.

### Phytoextraction indices

To measure the capacity of BB to reduce the HMs uptake in a *Brassica* shoot, the bio-concentration factor (BCF), translocation factor (TF) and metal extraction amount (MEA) were calculated^17, 20^. The percentage of immobilization of HMs (IM) after the incorporation of bamboo biochar was calculated by the following equation:

1 $$\mathrm{Bio-concentration}\,{\rm{Factor}}\,({\rm{BCF}})=\frac{{\rm{Metal}}\,{\rm{concentration}}\,{\rm{in}}\,{\rm{plant}}\,{\rm{tissue}}}{{\rm{Metal}}\,{\rm{concentration}}\,{\rm{in}}\,{\rm{soil}}}.$$

2 $${\rm{Translocation}}\,{\rm{Factor}}\,{\rm{TF}}=\frac{{\rm{Metal}}\,{\rm{concentration}}\,{\rm{in}}\,{\rm{shoot}}}{{\rm{Metal}}\,{\rm{concentration}}\,{\rm{in}}\,{\rm{root}}}.$$

3 $${\rm{Metal}}\,{\rm{Extraction}}\,{\rm{Amount}}\,({\rm{MEA}})={\rm{Metal}}\,{\rm{concentration}}\,{\rm{in}}\,{\rm{aerial}}\,{\rm{parts}}\times \mathrm{Biomass}\,$$

4 $$\begin{array}{l}{\rm{IM}}\,( \% )=\frac{DTPA\,extractable\,HMs\,in\,control-DTPA\,extractable\,HMs\,in\,treated\,samples}{DTPA\,extractable\,HMs\,in\,control}\,\times \,100.\end{array}$$

### Quality control and statistical analysis

The experiment was carried out in triplicate. Reagent blanks were used to correct the analytical values. Standard reference materials for wheat (GBW10011) and soil (GBW07405) were obtained from the National Research Center of Certified Reference Materials (Beijing, China). The recovery of the standard wheat sample ranged from 95.3–100.2, 97.7–106, 95.9–103 and 96.2–103.5% for Cd, Cu, Pb and Zn, respectively. The recovery of the standard soil sample ranged from 91.5–103.2%, 96.7–107.5%, 95.3–105.7% and 92.3–104.5% for Cd, Cu, Pb and Zn, respectively. The results of the standard wheat and soil samples were acceptable. All the experimental data were subjected to one-way ANOVA (*p* < 0.05) for independent variable analysis using IBM SPSS Statistics 22.0. All graphs were prepared in Origin-Pro (version 7.5).

## References

[1] Shahid M (2017). Foliar heavy metal uptake, toxicity and detoxification in plants: A comparison of foliar and root metal uptake. Journal of Hazardous Materials.

[2] Cheng J, Qiu H, Chang Z, Jiang Z, Yin W (2016). The effect of cadmium on the growth and antioxidant response for freshwater algae Chlorella vulgaris. Springerplus.

[3] Hua M (2012). Heavy metal removal from water/wastewater by nanosized metal oxides: A review. Journal of Hazardous Materials s.

[4] Alvarez A (2017). Actinobacteria: Current research and perspectives for bioremediation of pesticides and heavy metals. Chemosphere.

[5] Fiaz K (2014). Drought impact on Pb/Cd toxicity remediated by biochar in Brassica campestris. Journal of Soil Science & Plant Nutrition.

[6] Xu P (2016). The effect of biochar and crop straws on heavy metal bioavailability and plant accumulation in a Cd and Pb polluted soil. Ecotoxicol Environ Saf.

[7] Abujabhah IS, Bound SA, Doyle R, Bowman JP (2016). Effects of biochar and compost amendments on soil physico-chemical properties and the total community within a temperate agricultural soil. Applied Soil Ecology.

[8] Kim, H. S. *et al*. Effect of biochar on reclaimed tidal land soil properties and maize (Zea mays L.) response. *Chemosphere* **142**, doi:10.1016/j.chemosphere.2015.06.041 (2016). 10.1016/j.chemosphere.2015.06.041 26138709

[9] Xian Y, Wang M, Chen W (2015). Quantitative assessment on soil enzyme activities of heavy metal contaminated soils with various soil properties. Chemosphere.

[10] Li Y (2015). Phytotoxicity assessment on corn stover biochar, derived from fast pyrolysis, based on seed germination, early growth, and potential plant cell damage. Environmental Science & Pollution Research.

[11] Wang Y (2014). Effects of biochar on photosynthesis and antioxidative system of Malus hupehensis Rehd. seedlings under replant conditions. Scientia Horticulturae.

[12] Mahar A (2016). Impact of CaO, fly ash, sulfur and Na2S on the (im)mobilization and phytoavailability of Cd, Cu and Pb in contaminated soil. Ecotoxicology and Environmental Safety.

[13] Hmid A, Chami ZA, Sillen W, Vocht AD, Vangronsveld J (2015). Olive mill waste biochar: a promising soil amendment for metal immobilization in contaminated soils. Environmental Science and Pollution Research.

[14] Wang Q (2016). Evaluation of medical stone amendment for the reduction of nitrogen loss and bioavailability of heavy metals during pig manure composting. Bioresource Technology.

[15] Ahmad M (2016). Lead and copper immobilization in a shooting range soil using soybean stover- and pine needle-derived biochars: Chemical, microbial and spectroscopic assessments. Journal of Hazardous Materials.

[16] Marchand L (2016). Trace element bioavailability, yield and seed quality of rapeseed (Brassica napus L.) modulated by biochar incorporation into a contaminated technosol. Chemosphere.

[17] Lu H (2014). Can Biochar and Phytoextractors Be Jointly Used for Cadmium Remediation?. Plos One.

[18] Mackie KA, Marhan S, Ditterich F, Schmidt HP, Kandeler E (2015). The effects of biochar and compost amendments on copper immobilization and soil microorganisms in a temperate vineyard. Agriculture Ecosystems & Environment.

[19] Roberts, D. A., Paul, N. A., Dworjanyn, S. A., Bird, M. I. & Nys, R. D. Biochar from commercially cultivated seaweed for soil amelioration. *Scientific Reports* **5**, doi:10.1038/srep09665 (2015). 10.1038/srep09665 PMC4391317 25856799

[20] Kim HS (2015). Effect of biochar on heavy metal immobilization and uptake by lettuce (Lactuca sativa L.) in agricultural soil. Environmental Earth Sciences.

[21] Xu ZY, Tang M, Chen H, Ban YH, Zhang HH (2012). Microbial community structure in the rhizosphere of Sophora viciifolia grown at a lead and zinc mine of northwest China. Science of the Total Environment.

[22] Ma SC (2015). Effects of mine wastewater irrigation on activities of soil enzymes and physiological properties, heavy metal uptake and grain yield in winter wheat. Ecotoxicol Environ Saf.

[23] Mobin M, Khan NA (2007). Photosynthetic activity, pigment composition and antioxidative response of two mustard (Brassica juncea) cultivars differing in photosynthetic capacity subjected to cadmium stress. Journal of Plant Physiology.

[24] Al-Wabel MI (2014). Conocarpus biochar as a soil amendment for reducing heavy metal availability and uptake by maize plants. Saudi Journal of Biological Sciences.

[25] Lu, K. *et al*. Effect of bamboo and rice straw biochars on the mobility and redistribution of heavy metals (Cd, Cu, Pb and Zn) in contaminated soil. *Journal of Environmental Management*, In Press, doi:10.1016/j.jenvman.2016.05.068 (2016). 10.1016/j.jenvman.2016.05.068 27264699

[26] Houben D, Evrard L (2013). & Sonnet, P. Mobility, bioavailability and pH-dependent leaching of cadmium, zinc and lead in a contaminated soil amended with biochar. Chemosphere.

[27] Shen X (2015). Phytoavailability of Cd and Pb in crop straw biochar-amended soil is related to the heavy metal content of both biochar and soil. Journal of Environmental Management.

[28] Yang X (2016). Effect of biochar on the extractability of heavy metals (Cd, Cu, Pb, and Zn) and enzyme activity in soil. Environmental Science and Pollution Research.

[29] Fellet G, Marchiol L, Vedove GD, Peressotti A (2011). Application of biochar on mine tailings: Effects and perspectives for land reclamation. Chemosphere.

[30] Ahmad M (2014). Biochar as a sorbent for contaminant management in soil and water: A review. Chemosphere.

[31] Usman ARA (2013). Chemically Modified Biochar Produced from Conocarpus Wastes: An Efficient Sorbent for Fe(II) Removal from Acidic Aqueous Solutions. Adsorption Science & Technology.

[32] Zheng RL (2012). The effects of biochars from rice residue on the formation of iron plaque and the accumulation of Cd, Zn, Pb, As in rice (Oryza sativa L.) seedlings. Chemosphere.

[33] Lucchini P, Quilliam RS, Deluca TH, Vamerali T, Jones DL (2014). Increased bioavailability of metals in two contrasting agricultural soils treated with waste wood-derived biochar and ash. Environmental Science and Pollution Research.

[34] Kazemi N, Khavari-Nejad RA, Fahimi H, Saadatmand S, Nejad-Sattari T (2010). Effects of exogenous salicylic acid and nitric oxide on lipid peroxidation and antioxidant enzyme activities in leaves of Brassica napus L. under nickel stress. Scientia Horticulturae.

[35] Cenkci S (2010). Lead contamination reduces chlorophyll biosynthesis and genomic template stability in Brassica rapa L. Environmental & Experimental Botany.

[36] Kotroczó Z (2014). Soil enzyme activity in response to long-term organic matter manipulation. Soil Biology & Biochemistry.

[37] Pathan SI (2017). Seasonal variation and distribution of total and active microbial community of β-glucosidase encoding genes in coniferous forest soil. Soil Biology and Biochemistry.

[38] Lehmann J (2011). Biochar effects on soil biota – A review. Soil Biology and Biochemistry.

[39] Chang EH, Wang CH, Chen CL, Chung RS (2014). Effects of long-term treatments of different organic fertilizers complemented with chemical N fertilizer on the chemical and biological properties of soils. Soil Science & Plant Nutrition.

[40] Ding (2007). Physiological basis of different allelopathic reactions of cucumber and figleaf gourd plants to cinnamic acid. Journal of Experimental Botany.

[41] Reddy MK, Alexanderlindo RL, Nair MG (2005). Relative inhibition of lipid peroxidation, cyclooxygenase enzymes, and human tumor cell proliferation by natural food colors. Journal of Agricultural & Food Chemistry.

[42] Shen F (2017). Spatial distribution and risk assessment of heavy metals in soil near a Pb/Zn smelter in Feng County, China. Ecotoxicology and Environmental Safety.

[43] Hu X-F (2014). Effects of mining wastewater discharges on heavy metal pollution and soil enzyme activity of the paddy fields. Journal of Geochemical Exploration.

[44] Chen Y-X (2010). Effects of bamboo charcoal and bamboo vinegar on nitrogen conservation and heavy metals immobility during pig manure composting. Chemosphere.

[45] Ahammed GJ (2013). Role of brassinosteroids in alleviation of phenanthrene–cadmium co-contamination-induced photosynthetic inhibition and oxidative stress in tomato. Journal of Experimental Botany.

